# Inhibitor of serine peptidase 2 enhances *Leishmania major* survival in the skin through control of monocytes and monocyte-derived cells

**DOI:** 10.1096/fj.201700797R

**Published:** 2017-11-16

**Authors:** Amy Goundry, Audrey Romano, Ana Paula C. A. Lima, Jeremy C. Mottram, Elmarie Myburgh

**Affiliations:** * Wellcome Centre for Molecular Parasitology, Institute of Infection, Immunity, and Inflammation, College of Medical, Veterinary, and Life Sciences, University of Glasgow, Glasgow, United Kingdom;; † Department of Biology, Centre for Immunology and Infection, University of York, York, United Kingdom;; ‡ Instituto de Biofísica Carlos Chagas Filho, Universidade Federal do Rio de Janeiro, Rio de Janeiro, Brazil

**Keywords:** inflammatory monocytes, parasite, dermis, leishmaniasis, *in vivo*

## Abstract

*Leishmania major* is the causative agent of the neglected tropical disease, cutaneous leishmaniasis. In the mouse, protective immunity to *Leishmania* is associated with inflammatory responses. Here, we assess the dynamics of the inflammatory responses at the lesion site during experimental long-term, low-dose intradermal infection of the ear, employing noninvasive imaging and genetically modified *L. major*. Significant infiltrates of neutrophils and monocytes occurred at 1–4 d and 2–4 wk, whereas dermal macrophage and dendritic cell (DC) numbers were only slightly elevated in the first days. Quantitative whole-body bioluminescence imaging of myeloperoxidase activity and the quantification of parasite loads indicated that the *Leishmania* virulence factor, inhibitor of serine peptidase 2 (ISP2), is required to modulate phagocyte activation and is important for parasite survival at the infection site. ISP2 played a role in the control of monocyte, monocyte-derived macrophage, and monocyte-derived DC (moDC) influx, and was required to reduce iNOS expression in monocytes, monocyte-derived cells, and dermal DCs; the expression of CD80 in moDCs; and levels of IFN-γ *in situ.* Our findings indicate that the increased survival of *L. major* in the dermis during acute infection is associated with the down-regulation of inflammatory monocytes and monocyte-derived cells *via* ISP2.—Goundry, A., Romano, A., Lima, A. P. C. A., Mottram, J. C., Myburgh, E. Inhibitor of serine peptidase 2 enhances *Leishmania major* survival in the skin through control of monocytes and monocyte-derived cells.

*Leishmania* spp. are protozoan parasites that cause a spectrum of pathologies, ranging from skin ulceration to visceral dissemination, depending on the parasite species and the genetic background of the host, in humans and other vertebrates. *Leishmania major* is the major cause of cutaneous leishmaniasis, the most common manifestation of leishmaniases in humans. *Leishmania* parasites are transmitted to the host dermis as infective metacyclic promastigotes by an infected phlebotomine sand fly during a blood meal. Tissue damage caused by the proboscis of the sand fly, in addition to sand fly and *Leishmania*-derived factors, leads to the initiation of a strong local inflammatory immune response at the site of inoculation (1–3). After intradermal infection in mice, there is coordinated recruitment of innate immune cells: neutrophils are the first responders, arriving within only a few hours to become the first host cells for intracellular *Leishmania* (2, 4–6), whereas monocytes and macrophages infiltrate around 2–3 d to become the main host cells for persistent productive infection (5, 6). Although the initial cellular infiltrates likely result from a response to the injury provoked by the infection—either by needle injection or sand fly bites—later waves of recruitment are thought to depend directly on parasitism (2, 6). Dendritic cells (DCs), particularly monocyte-derived DCs (moDCs), are also recruited to the infection site and are important for the priming of antigen-specific T helper (T_h_) adaptive immune responses (7–9). In the mouse model, the IL-12–driven T_h_1 response is considered to be essential for protection against *L. major* infection; T_h_1 cells secrete IFN-γ, which stimulates the expression of iNOS, the enzyme that is responsible for the generation of NO (10).

Of importance, there is growing evidence that early parasite uptake by neutrophils is crucial for establishing infection in the dermis *via* down-regulation of T_h_1 immune response (6, 11). Engulfment of infected neutrophils by DCs that were recruited to an *L. major* infection site not only reduced DC activation and antigen presentation, but also inhibited the T_h_1 response in the next 2 wk (6). Similar observations using *Leishmania mexicana* have provided evidence that neutrophils contribute largely to block both the development of a protective immune response and the control of lesion progression (11). These studies have contributed to our knowledge of the dynamics of the initial steps of innate responses and how this affects parasitism; however, systematic *in vivo* studies of the dynamics of cellular populations at the lesion site and their responses at later chronic stages (*i.e.*, after 2 wk) are still lacking. Furthermore, technical limitations for real-time assessments of parasites and infiltrating cells, to date, have precluded systematic *in vivo* analyses using low-dose infections, which are thought to better represent the low-dose inoculation of sand fly–transmitted parasites and the ensuing immune response (12–14).

*Leishmania* employ virulence factors to alter cell signaling pathways in neutrophils, macrophages, and DCs, thereby facilitating the initiation and persistence of infection. Modulation of host signaling cascades may affect cytokine/chemokine production, cellular recruitment and activation, and the ensuing adaptive immune response, and may ultimately lead to the suppression of cell effector functions that are required for parasite killing (15). Despite much being known about the immune responses to *Leishmania* spp., roles of only a few putative virulence factors have been characterized with respect to the modulation of host immune responses *in vivo*. For example, it is known that *L. major* induces a counterprotective T_h_2 response *via* their surface lipophosphoglycan and other phosphoglycans, which inhibit the production of IL-2, IL-12, and IFN-γ, and stimulate the production of IL-4 and IL-10 (16). Surface protease GP63 of *L. major* is able to cleave multiple host proteins, including protein tyrosine phosphatases, which leads to the down-regulation of proinflammatory responses and antigen presentation (17); however, how lipophosphoglycan and GP63 affect cellular recruitment and activation in the skin over time remains unknown.

We have recently identified another *Leishmania* virulence factor, termed inhibitor of serine peptidase 2 (ISP2), that is important for the establishment of murine macrophage infections *in vitro* (18, 19). ISP2 is one of 3 orthologs of the bacterial protease inhibitor, ecotin, which inactivates serine peptidases from the S1A family, including neutrophil elastase (NE), cathepsin G, and proteinase 3 (20, 21). Serine peptidases regulate inflammatory responses *via* the proteolytic cleavage of cytokines, chemokines, and cell receptors (22, 23). In mammals, their activity is tightly regulated by naturally occurring peptidase activators or inhibitors, such as serpins, and the disruption of the peptidase-inhibitor balance can lead to the dysregulation of inflammatory responses, as is observed in chronic obstructive pulmonary disease (24–26) and acute experimental arthritis (27).

We have previously shown that *L. major* metacyclic promastigotes that are deficient in ISP2 and ISP3 (Δ*isp2/3*) are killed more efficiently by murine macrophages after their internalization, and that those remaining display delayed intracellular development (19). *Leishmania* lacks genes that encode S1A serine peptidases, and ISP2 has been shown to be a potent inhibitor of NE (18, 28), a peptidase that is found in the azurophilic granules of neutrophils and on the surface of monocytes and macrophages. Inhibition of NE activity by ISP2 during *Leishmania–*macrophage interaction *in vitro* prevents the activation of TLR4-mediated responses, including reactive oxygen species production, favoring parasite survival and intracellular development (19). More recently, the enhanced killing of Δ*isp2/3* by macrophages was shown to be dependent on TLR2, TLR4, and CD11b; the adaptor proteins MyD88 and TRIF; and protein kinase R, a double-stranded RNA-sensing kinase (29). ISP2-mediated modulation of phagocytosis was also linked to the kinin pathway, with the suggestion that ISP limits proinflammatory kinin release *via* its inhibition of surface peptidases during the initial parasite uptake (30).

We next asked how the infiltration and activation of immune cell populations develop over time (*i.e.*, up to 10 wk) during *L. major* infection *in vivo*, and whether ISP2 can influence these cellular dynamics, affecting local parasitism. In this study, noninvasive, real-time *in vivo* imaging of myeloperoxidase (MPO) activity, in combination with longitudinal flow cytometry, enabled the kinetic analysis of cellular populations and their activation state in the mouse ear dermis in a long-term, low-dose infection. The role of ISP2 in host cell recruitment, activation, and, ultimately, parasite survival *in vivo* was assessed. We propose that recruited monocytes are major players in the control of *L. major* infection of the skin, and that the infiltration and activation of these cells is modulated by ISP2 to facilitate parasite survival at the site of inoculation.

## MATERIALS AND METHODS

### Ethics statement

All animal procedures adhered to experimental guidelines and were approved by the United Kingdom Home Office and the University of Glasgow Ethics Committee under Project License 60/4442.

### Parasites and infection of mice

*L. major* Friedlin (MHOM/JL/80/Friedlin) were grown as promastigotes in modified Eagle’s medium, designated HOMEM (Thermo Fisher Scientific, Waltham, MA, USA), that was supplemented with 10% heat-inactivated fetal bovine serum (FBS, Thermo Fisher Scientific) and incubated at 25°C. Parasite lines that were deficient in *ISP2* and *ISP3* (Δ*isp2/3*) and that reexpressed *ISP2* and *ISP3* (Δ*isp2/3:ISP2/3*) were generated by gene replacement and reintroduction, as described previously (18). Promastigotes of Δ*isp2/3* and Δ*isp2/3:ISP2/3* grew similarly to *L. major* wild type (WT) *in vitro* (Supplemental Fig. 1*A*). Cell lines that overexpressed *ISP2* [WT (*pXG-ISP2*)] were generated *via* the introduction of an episomal copy of *ISP2* into *L. major* WT (28). Metacyclic promastigotes were isolated from a stationary phase culture by agglutination of other promastigote forms with peanut lectin, as previously described (31). Lesion-derived amastigotes were purified as previously described (32). Female C57BL/6J mice (8–14 wk old; Charles River Laboratories, Wilmington, MA, USA) were inoculated with 10^4^ metacyclic promastigotes in 10 μl PBS into the ear dermis.

### Bioluminescence imaging

Mice were imaged under isoflurane anesthesia in an IVIS Spectrum *in vivo* imaging system (PerkinElmer, Waltham, MA, USA) 10–15 min after i.p. injection of the substrate, luminol sodium salt (Sigma-Aldrich, St. Louis, MO, USA), in PBS at 200 mg/kg body weight. Images were acquired with an open emission filter, 1-min exposure, large binning, and 1 f/stop, and were captured with a charge-coupled device camera. Analysis was performed by using Living Image software (PerkinElmer). The absolute unit of photon emission was given as radiance (photons per second per square centimeter per steradian). Regions of interest were manually selected over the entire ear to quantify the amount of photon emission as total photon flux in photons per second.

### Limiting dilution assays

Ears were soaked in 70% ethanol for 5 min, air dried, and deposited in DMEM/2% FBS. Ears were cut repeatedly with surgical scissors and digested with 2.5 mg/ml collagenase D (Roche, Basel, Switzerland) while shaking at 37°C for 2 h. Digested ear tissue was mechanically dissociated with the back of a syringe through a 70-μm cell strainer (BD Biosciences, San Jose, CA, USA), washed, and centrifuged at 3000 *g* for 8 min. Draining retromaxillary lymph nodes (dLNs) were mechanically dissociated with the back of a syringe through a 70-μm cell strainer (BD Biosciences). Quantification of parasite burdens was performed by using the limiting dilution assay method (33). Ear and dLN homogenates were resuspended in HOMEM that was supplemented with 20% FBS, 100 U/ml penicillin, 100 µg/ml streptomycin, and 50 µg/ml gentamycin (Roche), and serially diluted in duplicate in 96-well flat-bottom plates. Plates were incubated in a humidified box at 25°C for 7–10 d, after which wells were visually analyzed weekly for 3 wk for the highest dilution well with live parasites. Parasite numbers are given as total per tissue or organ.

### Flow cytometry

Ears were deposited in PBS, cut repeatedly with surgical scissors, and digested with 4 mg/ml collagenase D (Roche) and 100 U/ml DNase I at 37°C for 45 min while shaking. Digested tissue was processed in a gentleMACS dissociator (Miltenyi Biotec, Bergisch Gladbach, Germany) and filtered through a 40-μm cell strainer (BD Biosciences). dLNs were mechanically dissociated with the back of a syringe through a 70-μm cell strainer (BD Biosciences). Homogenized tissue and dLN samples were centrifuged at 380 *g* for 10 min at 4°C, washed, and resuspended in PBS. All additional steps were performed at 4°C and centrifugation was performed at 380 *g* for 5 min. Cells were blocked with anti-Fcγ III/II (CD16/32) receptor Ab (2.4G2; BD Biosciences) for 30 min, then stained with fluorochrome-conjugated Abs for 30 min. The following anti-mouse Abs were used: APC-CD11b (M1/70), PE-Cy7-CD11b (M1/70), PE-CD11c (N418), PerCP-Cy5.5-CD11c (N418), FITC-CD80 (16-10A1), PE-Cy7-CD86 (GL1), eFluor450-Ly-6C (HK1.4), PerCP-Cy5.5-Ly-6C (HK1.4), eFluor450-MHCII (M5/114.15.2), FITC-MHCII (M5/114.15.2), and PE-NOS2 (CXNFT; Thermo Fisher Scientific); and APC-eFluor780-Ly-6G (1A8), PE-Ly-6G (1A8), and FITC-MHCII (2G9) from BD Biosciences. Isotype controls used were rat IgG2a, rat IgG2b, rat IgG2c, or Armenian hamster IgG. After staining of surface markers, cells were washed twice with PBS, stained with Fixable Viability Dye eFluor506 or eFluor660 (Thermo Fisher Scientific) for 30 min according to manufacturer protocol, and fixed with methanol-free formaldehyde (Thermo Fisher Scientific) for 5 min. For staining of cytoplasmic intracellular antigens, cells were pulse vortexed, fixed in 100 μl IC fixation buffer (Thermo Fisher Scientific) for 20 min, washed in 1× permeabilization buffer (Thermo Fisher Scientific), and stained for 20 min. Cells were resuspended in flow-cytometry buffer (1% dialyzed FBS, 0.05% sodium azide, 2 mM EDTA, in PBS) and passed through a Nitex mesh with a pore size of 50 μm (Cadisch, Hatfield, United Kingdom). Data were collected by using either a MACSQuant Analyzer (Miltenyi Biotec) or a BD LSR II Flow Cytometer (BD Biosciences), and were analyzed by using FlowJo (Tree Star, Ashland, OR, USA). Compensation settings were optimized by using lymph node cells that were single stained with anti-mouse CD4 Abs conjugated to the corresponding fluorophores used (RM4-5; Thermo Fisher Scientific; and GK1.5; BD Biosciences). Live—on the basis of Fixable Viability Dye staining—innate immune cells from the ear and dLNs were identified on the basis of size (forward scatter) and granularity (side scatter), as well as by surface phenotype as indicated in the text and figure legends.

### ELISA and Luminex assays

Ears and lymph nodes were deposited in Tissue Protein Extraction Reagent (Thermo Fisher Scientific) with 1% protease inhibitor cocktail (Roche). Lysis was performed on a TissueLyser LT (Qiagen, Germantown, MD, USA) with two 5-mm stainless steel beads (Roche) at 50 Hz for 2 min. Samples were centrifuged at 13,000 rpm for 10 min at 4°C, and supernatants were stored at −80°C. Mouse IFN-γ ELISA Ready-Set-Go! Kit (Thermo Fisher Scientific) was used according to manufacturer protocol. Plates were read on a Dynex MRX TC II microplate reader (Dynex Technologies, Chantilly, VA, USA) at 450 nm. Bio-Plex Pro Mouse 23-plex Assay (Bio-Rad, Hercules, CA, USA) was used according to manufacturer protocol, and plates were read on a Luminex 100 plate reader (Luminex, Austin, TX, USA).

### Statistical analysis

All statistical analyses were performed with Prism (GraphPad Software, La Jolla, CA, USA) using the test described in the figure legends. Statistically significant differences (*P* < 0.05) are annotated on the graphs using symbols as described in the figure legends; differences that are not significant (*P* > 0.05) are not annotated. A table is included to summarize the significance level for parasite burden data.

## RESULTS

### ISP2 is important for parasite survival in the skin

**Figure 1.**
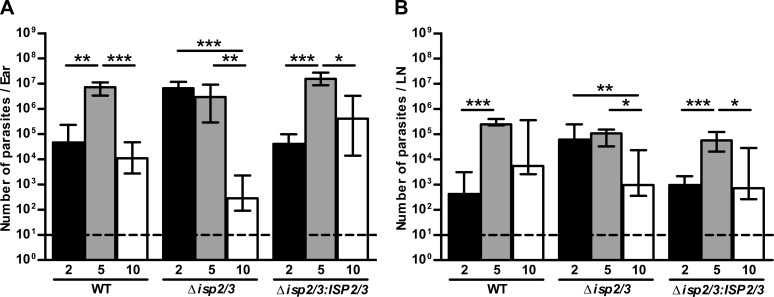
Parasite burdens during *L. major* infection. C57BL/6 mice were inoculated in the ear with 10^4^ *L. major* WT, Δ*isp2/3*, and Δ*isp2/3:ISP2/3* metacyclic promastigotes. Parasite loads in the ear (*A*) and dLN (*B*) at 2, 5, and 10 wk postinfection were determined by limiting dilution assay. Dashed lines indicate the lowest number of living parasites that could be detected among cellular debris. Medians and interquartile range of data from 2 independent experiments are shown (*n* = 9–11/group/time point). Table 1 summarizes the level of significance between groups for each time point. Asterisks indicate statistical significance within the groups. *P < 0.05, **P < 0.01, ***P < 0.001 (Kruskal Wallis with Dunn’s posttest).

To assess how ISP2 affects the infection dynamics of *L. major*, a low-dose intradermal ear infection model in C57BL/6 mice was used. Limiting dilution assays were performed to quantify *L. major* WT, Δ*isp2/3*, and Δ*isp2/3:ISP2/3* in infected ears and dLNs after 2, 5, and 10 wk. At 2 wk, Δ*isp2/3* parasite burdens in ears were significantly higher than WT and Δ*isp2/3:ISP2/3* (**Fig. 1*A*** and **Table 1**). Δ*isp2/3* burdens remained unchanged by 5 wk, whereas WT and Δ*isp2/3:ISP2/3* had increased compared with levels at 2 wk, with more Δ*isp2/3:ISP2/3* compared with Δ*isp2/3*. By 10 wk, Δ*isp2/3* burdens had decreased significantly compared with those at 2 and 5 wk, whereas burdens of WT and Δ*isp2/3:ISP2/3* had reduced to the 2-wk levels. In addition, Δ*isp2/3* burdens in the ear were significantly lower (∼1000-fold) at 10 wk compared with Δ*isp2/3:ISP2/3.* A similar trend was observed for dLNs, with significantly more Δ*isp2/3* parasites than WT and Δ*isp2/3:ISP2/3* at 2 wk and a 100-fold reduction in Δ*isp2/3* parasite loads by 10 wk compared with 2 and 5 wk. (Fig. 1*B* and Table 1). Overall, these data suggest that ISP2 is important for *L. major* survival at the site of infection; it contributes to the control of parasite load early in infection for enhanced survival at later stages of infection. The *ISP2/3* reexpressing line, which has a higher level of ISP2 than *L. major* WT (18), demonstrated slower parasite expansion early in infection, with significantly increased burdens compared with Δ*isp2/3* later. These observations suggest that the fine-tuning of ISP2 expression can have a major impact on skin infection in a physiologic model of infection.

**TABLE 1.** Significance level of differences between parasite groups at each time point

Parasite groups compared	*P*					
	Ear (wk)			Lymph node (wk)		
	2	5	10	2	5	10
WT *vs.* Δ*isp2/3*	<0.001	NS	NS	<0.001	<0.05	NS
Δ*isp2/3* *vs.* Δ*isp2/3*:*ISP2/3*	<0.001	<0.01	<0.01	<0.001	NS	NS
WT *vs.* Δ*isp2/3*:*ISP2/3*	NS	NS	NS	NS	<0.01	NS

Significance level was measured by Kruskal Wallis with Dunn’s posttest.

In addition, lesion development in ears was monitored weekly by using the Schuster scoring system (34). Δ*isp2/3*-infected ears developed lesions earlier than WT and Δ*isp2/3:ISP2/3* infections, with a mean score of 1.5 by 2 wk compared with 0.1 and 0.3 for WT and Δ*isp2/3:ISP2/3,* respectively (Supplemental Fig. 1*B*). Lesion development in the low-dose ear model has been shown to correspond with inflammation (12), which suggests that the presence of ISP2 may delay the onset of inflammation.

### ISP2 influences cellular recruitment and activation

**Figure 2.**
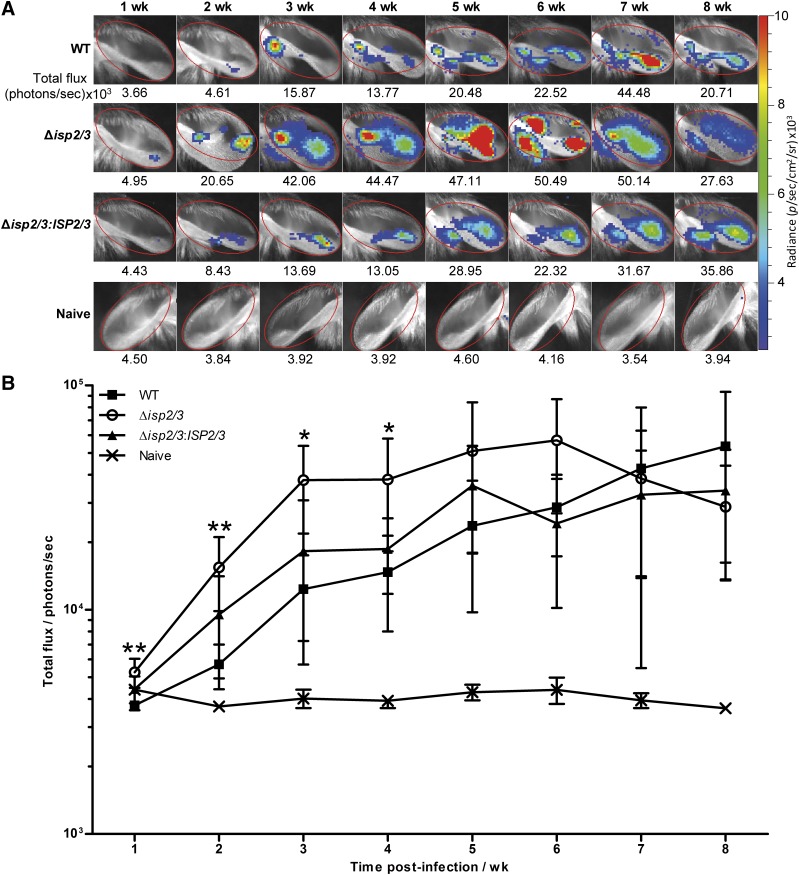
*In vivo* bioluminescence imaging of MPO activity at the inoculation site during the chronic stage of infection. C57BL/6 mice were inoculated in the ear with 10^4^ *L. major* WT, Δ*isp2/3*, and Δ*isp2/3:ISP2/3* metacyclic promastigotes. Mice were imaged 10–15 min after intraperitoneal luminol injection. *A*) Representative images for the group, closest to the mean, over the course of infection for ears that were infected with parasite cell lines (*n* = 5/group) and naive control ears (*n* = 4, left). The color scale indicates bioluminescence radiance in photons per second per square centimeter per steradian. The same color scale and region of interest (ROI; red oval) was applied to all images, and the total flux for each ROI is given beneath the image. *B*) Total flux for the group over 8 wk. Means ± sd are shown and data are representative of 2 independent experiments. Statistical significance between *L. major* WT and Δ*isp2/3* from 1–4 wk is indicated. *P < 0.05, **P < 0.01 (1-way ANOVA with a Tukey posttest).

We next went on to identify whether there were signs of increased inflammation in Δ*isp2/3*-infected ears. This was first investigated by imaging MPO activity of activated phagocytes *via* luminol-based bioluminescence (35) (**Fig. 2*A*** and Supplemental Fig. 2*A*). The ear dermis provides a good site for bioluminescence imaging, as the superficial surface foci minimizes light quenching to increase sensitivity. During the first 4 wk of infection, MPO-specific bioluminescence was significantly higher in Δ*isp2/3*-infected ears compared with WT (Fig. 2*B*). MPO-specific bioluminescence was also investigated in the initial stages of infection. At 1 h postinfection, low but significantly higher bioluminescence was detected in all *L. major–*infected ears compared with naive and PBS controls (Supplemental Fig. 2*B*); however, there were no significant differences between ears that were infected with the different parasite lines up to 48 h, by which time the signal decreased to background levels (Supplemental Fig. 2*C*). In addition, infection with a cell line that overexpressed ISP2, WT (*pXG-ISP2*) (28), demonstrated that an excess of ISP2 delayed MPO-specific bioluminescence, which increased only from 4 wk compared with 2 wk during WT and Δ*isp2/3* infection (Supplemental Fig. 2*D*). This suggests that the amount of ISP2 that is expressed by *L. major* may affect the activation status of innate cells at the site of infection, with higher ISP2 levels correlating with the containment of local inflammation.

### Increased recruitment of monocytic lineage cells to the site of *L. major* Δ*isp2/3* infection

**Figure 3.**
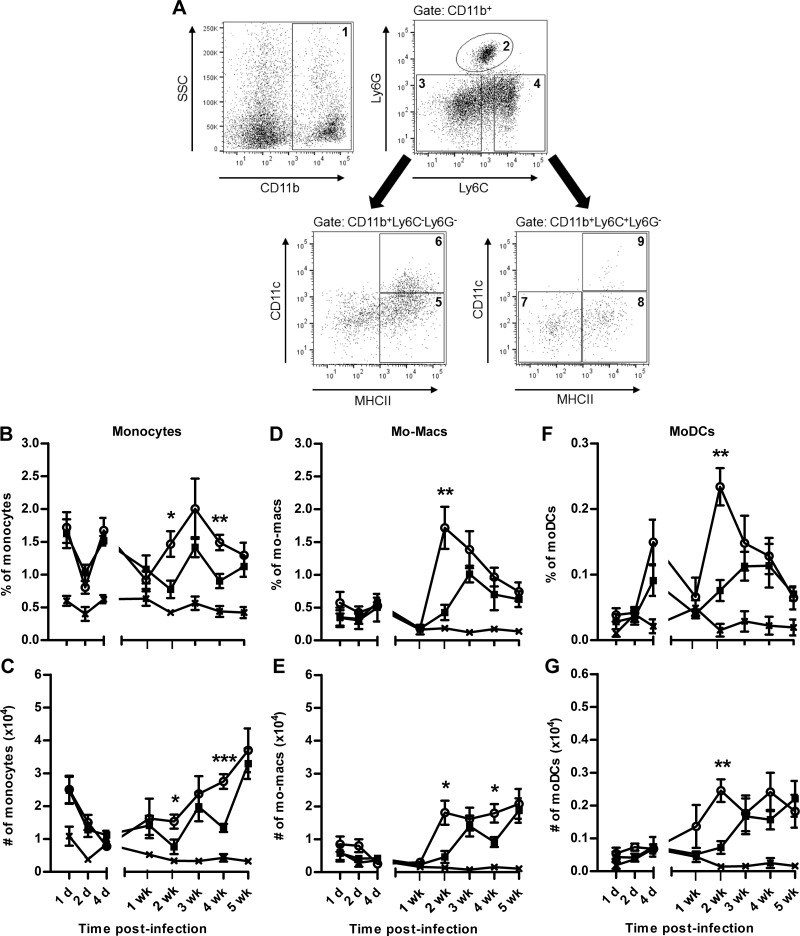
Dynamics of the innate immune cell populations at the site of *L. major* infection. C57BL/6 mice were inoculated in the ear with 10^4^ *L. major* WT and Δ*isp2/3* metacyclic promastigotes (*n* = 5/time point). *A*) Subpopulations of CD11b^+^ myeloid cells (region 1) were gated on Ly6C and Ly6G expression; regions 3 and 4 were further gated on CD11c and major histocompatibility complex (MHC)-II expression. The subpopulation of CD11b^+^ cells were defined as follows: region 2, neutrophils, Ly6C^int^Ly6G^+^; region 5, dermal macrophages, Ly6C^−^Ly6G^−^CD11c^−^MHCII^+^; region 6, dermal dendritic cells, Ly6C^−^Ly6G^−^CD11c^+^MHCII^+^; region 7, inflammatory monocytes, Ly6C^hi^Ly6G^−^CD11c^−^MHCII^−^; region 8, inflammatory macrophages, Ly6C^hi^Ly6G^−^CD11c^−^MHCII^+^; and region 9, inflammatory dendritic cells, Ly6C^hi^Ly6G^−^CD11c^+^MHCII^+^. *B*–*G*) Percentages within the CD11b^+^ population (top) and total number (bottom) of inflammatory monocytes (*B*, *C*), monocyte-derived macrophages (*D*, *E*), and moDCs (*F*, *G*) per ear during infection with *L. major* WT (▪) or Δ*isp2/3* (○). Naive ears from infected mice were used as a control at each time point (×). Results are expressed as means ± sem. Data are representative of 2 independent experiments. Asterisks indicate statistical significance between WT and Δ*isp2/3*. *P < 0.05, **P < 0.01, ***P < 0.001 (unpaired Student’s *t* test).

To investigate how the kinetics of innate cell populations develop during low-dose chronic infection—and whether ISP2 influences those events—we analyzed cellular composition after *L. major* WT or Δ*isp2/3* infection over 5 wk by using flow cytometry. For each animal, both *L. major*–infected and naive ears were analyzed. Neutrophils (Ly6C^int^Ly6G^+^), tissue-resident dermal macrophages (Ly6C^−^Ly6G^−^CD11c^−^MHCII^+^), dermal DCs (Ly6C^−^Ly6G^−^CD11c^+^MHCII^+^), inflammatory monocytes (Ly6C^hi^Ly6G^−^CD11c^−^MHCII^−^), monocyte-derived macrophages (Ly6C^hi^Ly6G^−^CD11c^−^MHCII^+^), and moDCs (Ly6C^hi^Ly6G^−^CD11c^+^MHCII^+^) were identified and quantified by the expression of surface markers within the CD11b^+^ myeloid cell population on the basis of a previous report (6) (**Fig. 3*A***).

The total CD11b^+^ population displayed a similar trend in WT and Δ*isp2/3*-infected ears, with comparable percentages of CD11b^+^ cells during the 5 wk of infection (Supplemental Fig. 3*A*), and a significantly higher total number of CD11b^+^ cells in Δ*isp2/3* infections at 2 d only (Supplemental Fig. 3*B*). However, we observed significant differences in individual cell types within this myeloid population, particularly in monocytes and monocyte-derived cells (Fig. 3*B–G*). Inflammatory monocytes were recruited to the ear within the first 2 d in both infections, followed by a decrease during the next 7–14 d and a second wave of infiltration, from 1 wk in Δ*isp2/3-*infected ears and from 2 wk in WT-infected ears (Fig. 3*B, C*). Similarly, monocyte-derived macrophages and moDCs increased within the first few days of infection, fell sharply to the level of a naive ear around 1 wk, and increased in the following weeks (Fig. 3*D–G*). A significantly higher proportion and total number of monocytes and monocyte-derived cells were observed in ears that were infected with Δ*isp2/3* parasites compared with those infected with WT.

Neutrophils were the predominant CD11b^+^ cells that were recruited to the ear 1 d after infection. The numbers then decreased to the levels of a naive ear until 2 wk, followed by a second wave, peaking at 4 wk. There were no significant differences in the proportions or total numbers of neutrophils between WT and Δ*isp2/3* infections (Supplemental Fig. 3*C*, *D*). Dermal macrophages and DCs also increased after infection, peaking at 2–4 d (Supplemental Fig. 3*E*–*H*), with a significantly higher number of dermal DCs in Δ*isp2/3*-infected ears at 2 d (Supplemental Fig. 3*H*).

Together, these data demonstrate that low-dose infections result in an increase in neutrophils, inflammatory monocytes, dermal DCs, and dermal macrophages in the first 4 d. This increase is initiated by neutrophils and small numbers of monocytes at 1 d and followed by DCs and macrophages, with all populations returning to naive levels in the first week. Another significant infiltration event starts around 2–3 wk, and increases continuously up to 5 wk, with neutrophils representing the cell type in highest proportion of the CD11b^+^ population. A similar recruitment pattern was observed previously in high-dose, intradermal *L. major* infections (3, 6), although these studies only assessed the cellular populations up to 2 wk postinfection. Of importance, in the absence of ISP2, neutrophil recruitment was unaffected, but the second wave of monocyte infiltration occurred earlier and those cells were also recruited at higher numbers. This was accompanied by an increase in the proportion of monocyte-derived macrophages and moDCs at the site of infection, which suggests that ISP2 influences both the kinetics and the intensity of monocyte infiltration and the yield of monocyte-derived cells at the lesion site.

### Increased iNOS expression in innate cells at the site of *L. major* Δ*isp2/3* infection

As we observed a significant reduction in parasite burdens in Δ*isp2/3-*infected ears between 2 and 10 wk (Fig. 1*A*), we assessed whether host protective responses against these *L. major* parasites were up-regulated. NO—generated by iNOS—is one of the critical microbicidal responses in the control of *L. major* infection (8, 9, 36, 37), and, thus, we evaluated the levels of iNOS expression by different cellular subsets at the infection site. We focused on 2 wk postinfection because of the greater differences in the cellular infiltrate between *L. major* WT and Δ*isp2/3* infections at this timepoint.

**Figure 4.**
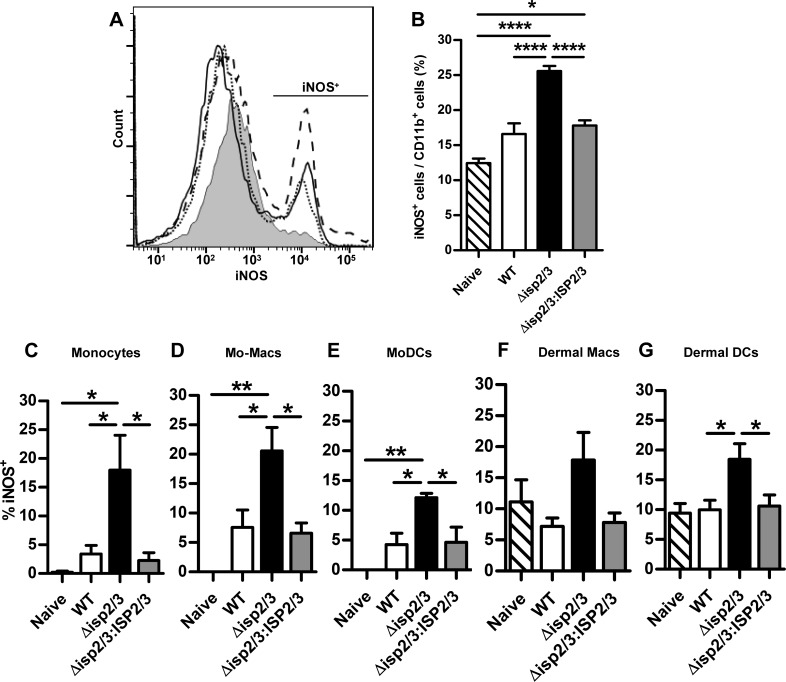
iNOS expression at the site of infection with *L. major*. C57BL/6 mice were inoculated in the ear with 10^4^ *L. major* WT, Δ*isp2/3*, and Δ*isp2/3:ISP2/3* metacyclic promastigotes (*n* = 5). Innate cell populations of the ear were analyzed by flow cytometry as described in Fig. 3. Data are presented as the percentage of iNOS^+^ cells within each cell type. *A*) Histogram shows iNOS^+^ cells within the CD11b^+^ population at 2 wk postinfection. Shaded area, naive; solid line, WT; dashed line, Δ*isp2/3*; dotted line, Δ*isp2/3:ISP2/3*. *B*) Percentage of iNOS^+^ cells within the CD11b^+^ population at 2 wk. *C*–*G*) Percentage of iNOS^+^ monocytes (*C*), monocyte-derived macrophages (*D*), moDCs (*E*), dermal macrophages (*F*), and dermal DCs (*G*), as assessed by flow cytometry. Means ± sem are shown. Data are representative of 2 independent experiments. Asterisks indicate statistical significance between groups. *P < 0.05, **P < 0.01, ****P < 0.0001 (1-way ANOVA with a Tukey posttest).

Flow cytometry revealed a significantly higher percentage of iNOS^+^ cells within the CD11b^+^ population in Δ*isp2/3*-infected ears compared with WT and Δ*isp2/3:ISP2/3* (**Fig. 4*A, B***). This higher iNOS response was a result of an increase in iNOS^+^ inflammatory monocytes, monocyte-derived macrophages, moDCs, and dermal DCs (Fig. 4*C–G*). The greater difference was observed in the percentage of iNOS-expressing monocytes and monocyte-derived macrophages, which was 4- to 5-fold higher in Δ*isp2/3*-infected ears. These data demonstrate that during infection with Δ*isp2/3*, both skin-resident phagocytes and those that are derived from recruited monocytes have higher microbicidal potential, but they are at low proportions (<10%) during infections with *L. major* WT.

### ISP2 delays DC maturation

**Figure 5.**
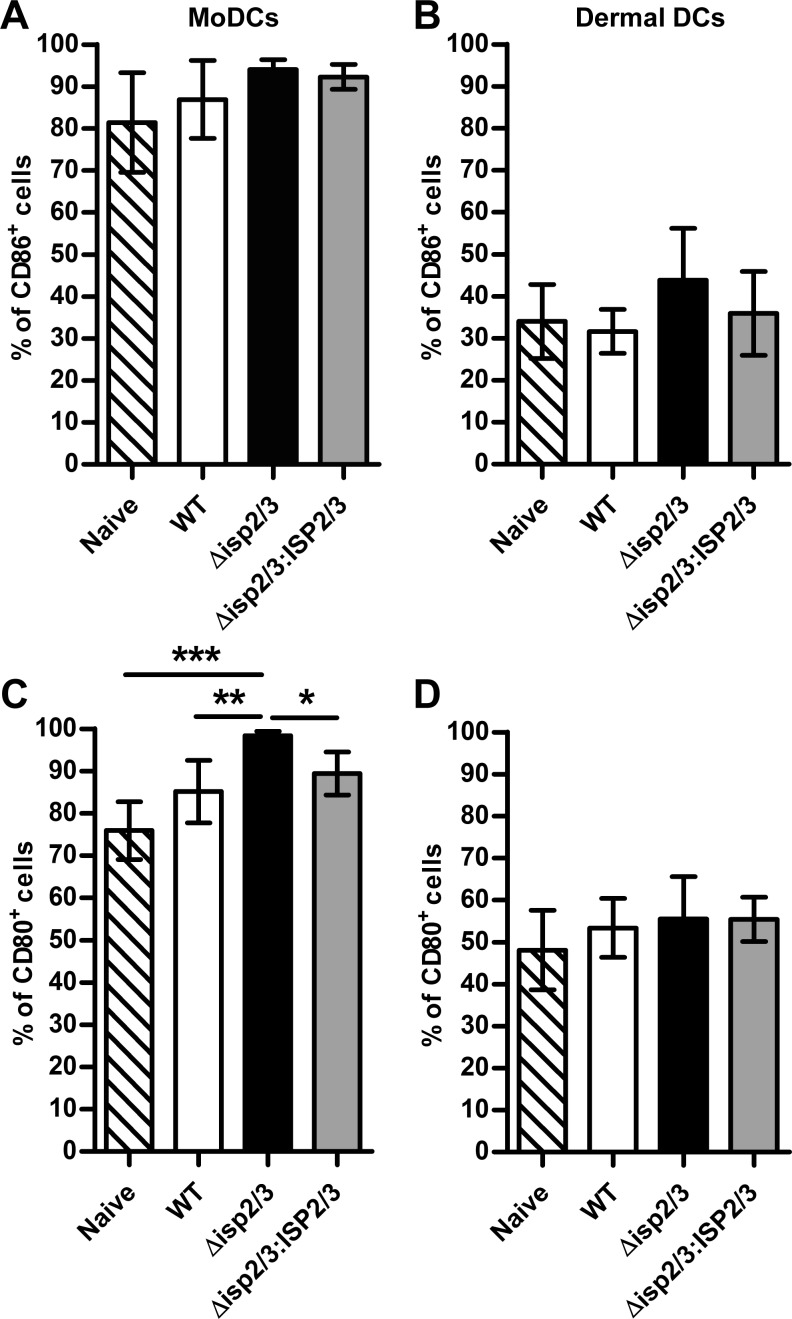
DC costimulatory molecule expression at 2 wk postinfection. C57BL/6 mice were inoculated in the ear with 10^4^ *L. major* WT, Δ*isp2/3*, and Δ*isp2/3:ISP2/3* metacyclic promastigotes (*n* = 7). Innate immune cell populations of the ear were analyzed by flow cytometry as shown in Fig. 3*A*. *A*, *B*) Percentage of CD86^+^ moDCs (*A*) and resident DCs (*B*). *C*, *D*) Percentage of CD80^+^ moDCs (*C*) and resident DCs (*D*). Results are expressed as means ± sem. Asterisks indicate statistical significance between groups. *P < 0.05, **P < 0.01, ***P < 0.001 (1-way ANOVA with a Tukey posttest).

DCs migrate from the site of *Leishmania* infection to the dLN where they present antigen to naive T cells to induce antigen-specific T-cell responses (7). We next investigated whether *L. major* ISP2 could influence DC activation to ultimately affect antigen presentation and parasite survival. Activation states of the dermal DC and moDC populations in the ear of infected mice were determined by examining their surface expression of the costimulatory molecules, CD86 and CD80, at 2 wk postinfection. CD86 expression was similar in DCs from naive control and WT-, Δ*isp2/3*-, and Δ*isp2/3:ISP2/3-*infected ears (**Fig. 5*A, B***); however, CD80 expression, a marker of mature DCs, was increased in moDCs in Δ*isp2/3*-infected ears compared with WT- and Δ*isp2/3:ISP2/3-*infected ears and naive control (Fig. 5*C*). CD80 expression on dermal DCs (Fig. 5*D*) was not significantly different between any of the groups. These data confirm that the presence of ISP2 was associated with the modulation of moDC maturation *in vivo*. In the absence of ISP2, up-regulation of DC costimulatory molecules enhances their T-cell stimulatory potential, which suggests that, in Δ*isp2/3* infection at this timepoint, DCs may be more primed for antigen presentation.

### Cytokine and chemokine responses in *L. major* WT and *ISP2* mutant infections

**Figure 6.**
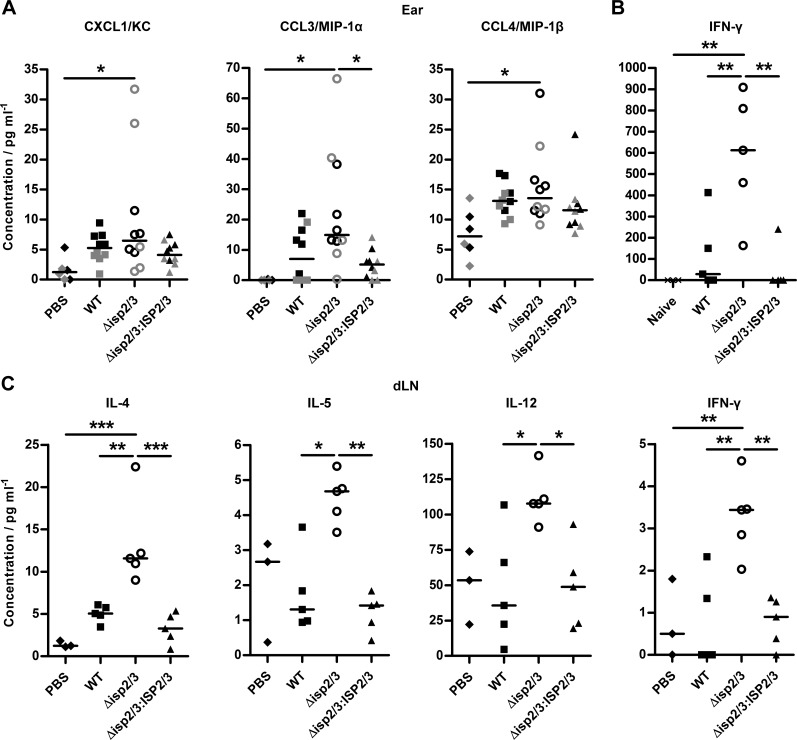
Cytokine responses at the site of infection and dLNs. C57BL/6 mice were inoculated in the ear with 10^4^ *L. major* WT, Δ*isp2/3*, and Δ*isp2/3:ISP2/3* metacyclic promastigotes (*n* = 5). *A*, *C*) A multiplex Luminex assay was performed by using ear (*A*) and dLN (*C*) samples of infected mice and PBS controls after 10 d. *B*) ELISA was performed to measure IFN-γ in infected ears and naive controls at 2 wk. Data from individual mice are shown and line indicates the median. Data are representative of 2 independent experiments. Asterisks indicate statistical significance between groups. *P < 0.05, **P < 0.01, ***P < 0.001 (1-way ANOVA with a Tukey posttest).

We next determined whether the absence of ISP2 was linked to alterations in cytokines and chemokines at the site of infection and in dLNs. A multiplex Luminex assay was performed on supernatants that were collected from ears and dLNs after 10 d of infection. Analysis of individual mice indicated the presence of 1–2 hyper-responsive mice in both experiments performed, which highlighted that the analysis of pooled samples or reporting of means only may skew results. Among tested chemokines, only CCL3 (MIP-1α) was found to be significantly higher in Δ*isp2/3-*infected ears compared with Δ*isp2/3:ISP2/3* (**Fig. 6*A***). CXCL1 (KC) and CCL4 (MIP-1β) were slightly elevated, but not significantly different. Furthermore, IFN-γ levels were measured by ELISA and found to be significantly higher in Δ*isp2/3*-infected ears at 2 wk compared with those of WT and Δ*isp2/3:ISP2/3* infections (Fig. 6*B*), which indicated an elevated T_h_1-type response and could explain the increased microbicidal response toward Δ*isp2/3* observed between 2 and 10 wk after infection.

In dLNs, IL-4, IL-5, IL-12, and IFN-γ were all significantly higher in Δ*isp2/3* infections compared with WT and Δ*isp2/3:ISP2/3* infections (Fig. 6*C*). Of note, IL-12 in dLNs were similar in mice that were injected with PBS or infected with WT or Δ*isp2/3:ISP2/3*, which is in agreement with the knowledge that *L. major* can reduce or block IL-12 production by macrophages and DCs (38, 39), whereas this ability was reduced in the absence of ISP2. This suggests that in infections with *L. major*, ISP2 contributes to delaying the induction of the adaptive immune response and, more specifically, attenuates the mounting of inflammatory responses.

## DISCUSSION

*Leishmania* inoculation into the skin induces the rapid infiltration of neutrophils that quickly ingest parasites and subsequently undergo apoptosis. Parasites are either phagocytosed within infected neutrophils by macrophages (40) and DCs (6), or are released for uptake by dermal macrophages and DCs, infiltrates of monocytes, and eventually monocyte-derived macrophages and moDCs (2, 41). These early interactions are crucial in shaping adaptive immune response and parasite killing. It is now well established that factors, such as the host genetic background, infection dose and site, and *Leishmania* species, impact these interactions (3, 9, 12, 42, 43). The latter suggests that parasite-derived factors play a role in this immune modulation and parasite survival; however, their study in this context has so far been lacking. We show here that ISP2 plays a role in the ability of *L. major* to establish infection and modulate monocytes and moDCs and monocyte-derived macrophages, thereby enhancing parasite survival in the skin. We previously reported that *Leishmania* ISP2 protects parasites against killing in macrophages *in vitro* by inhibiting NE activity and downstream NE-TLR4–mediated responses, including reactive oxygen species production (19). In this study, we specifically address the importance of ISP2 in the establishment of *Leishmania* infection *in vivo* in a low-dose intradermal C57BL/6 mouse model. We show higher early (2 wk) parasite loads in *L. major* Δ*isp2/3*-infected ears, followed by pronounced decrease in parasite load, which suggests enhanced parasite killing in the absence of ISP2. Whereas the reduction of the burden of Δ*isp2/3* in the ear at the chronic stage (between 5 and 10 wk) was 10,000-fold, this reduction was less pronounced in lymph nodes (100-fold), which suggests that lymph nodes could either receive a lower amount of infiltrating inflammatory cells or that long-term effects take place in a kinetics that differ from that at the infection site. In contrast, WT and the ISP2 reexpressor, Δ*isp2/3:ISP2/3*, which has been shown to overexpress ISP2 (18), had lower parasite numbers initially, increased significantly over the following weeks, and survived better by wk 10.

As the expression of ISP3 has not been detected in *L. major* procyclic or metacyclic promastigotes or amastigotes, the phenotypes that were observed with *ISP2/3* mutant cell lines are most likely a result of ISP2, which is abundantly expressed in WT (18).

These data demonstrate that *L. major* influences the second wave of cellular recruitment and activation in the ear dermis *via* ISP2. This second wave, which occurs at 1–2 wk postinfection, has been described in experimental *Leishmania* models (5, 6), but has yet to be fully characterized. Emphasis on the first wave of cellular infiltrates, in particular, neutrophils, in response to *Leishmania* infection has clearly established their importance in controlling disease (2, 6, 11). In this study, we demonstrate that ISP2 had no impact on the early recruitment of neutrophils and monocytes within the first week of infection, but it reduced inflammatory monocytes, monocyte-derived macrophages, and moDCs (also termed inflammatory DCs) (8) that were present in the ear between 2 and 4 wk. Early recruitment of neutrophils and monocytes is usually triggered by the secretion of chemokines, such as CXCL2 (MIP-2), CXCL8 (IL-8), CCL2 (MCP-1), and CCL3 (MIP-1α) from tissue-resident cells (44). This initial chemotactic response is further amplified by the incoming neutrophils that secrete proinflammatory cytokines and chemokines for more recruitment of neutrophils and monocytes. Infection studies that used CCL2^−/−^ and CCR2^−/−^ C57BL/6 mice suggested that CCL2 is dispensable for protection against *L. major*, but that another CCR2 ligand is required (45). CCL3, in contrast, enhances protection against *L. major* infection; *L. major* induces CCL3 secretion in infiltrating neutrophils, which is essential for the development of moDCs in the ear lesion (46). We observed an increase in CCL3 in ears that were infected with Δ*isp2/3* compared with Δ*isp2/3:ISP2/3,* which suggests that higher levels of ISP2 correlates with lower levels of CCL3. Of note, depletion of CCL3 has been observed to have no effect on neutrophil recruitment during the first 24 h after infection (46). On the basis of these findings and ours, we propose that ISP2 inhibits the activity of serine peptidases of host cells that are recruited early in infection, and that this interaction facilitates the changes we observed in CCL3 secretion and the second wave of cellular recruitment and activation.

The presence of ISP2 also reduced the activation of phagocytes in the ear and was associated with reduced IFN-γ and iNOS in the lesion, which suggests that ISP2 is important in limiting T_h_1-associated responses. IFN-γ has been reported to induce monocyte chemotaxis (47) and is required for the differentiation of monocytes into functional TNF-α– and iNOS-producing DCs (48). An increase in IFN-γ could also account for the increase in monocyte and monocyte-derived cells at the site of Δ*isp2/3* infection. Recruitment of inflammatory monocytes and their subsequent differentiation to moDCs has been proposed to be essential for the induction of protective T_h_1 responses against *L. major* (7). In agreement with this, *L. mexicana* infection, which induces a limited T_h_1 response and causes chronic lesions, recruits fewer monocytes with reduced development of moDCs (9). The data presented here support the notion that monocyte recruitment and moDC development contribute to the T_h_1-mediated killing of parasites in the skin/periphery. In addition, it highlights the importance of the second wave of cellular recruitment for parasite control and identifies its orchestrators as a major target for modulation by *Leishmania*.

*Leishmania* can directly modulate DC signaling and responses, such as their capacity to migrate, mature, present antigen, and produce cytokines (49, 50). In this study, we demonstrated that *L. major* inhibits the maturation of moDC *in vivo* *via* ISP2. We had previously established that ISP2 prevents the activation of a NE-TLR4 signaling cascade in macrophages (19). LPS-induced TLR4 signaling usually activates NF-κB (p65/p50) and AP-1 *via* a MyD88-dependent pathway for the production of proinflammatory cytokines, including TNF-α, IL-6, and IL-12p40 (51, 52), and chemokines, such as CXCL1 and CXCL2 (53). In addition, it has a MyD88-independent arm that activates interferon regulatory factor (IRF) 3 and IRF7 for the maturation of DCs, production of type I IFNs and IFN-stimulated genes, and induction of costimulatory molecules on monocytes (54, 55). TLR4 activation can also induce monocyte differentiation into macrophages and DCs (56). It is thus feasible that monocyte-derived macrophages and moDCs in *L. major* Δ*isp2/3* infection could be increased further as a result of TLR4-induced differentiation of monocytes, possibly *via* the absence of ISP2-mediated inhibition of serine peptidase activity.

We hypothesize that ISP2 exerts its effect on host serine peptidases during the initial entry into the host cell, with contact with host cells being important for ISP2-mediated modulation. Furthermore, as predicted target peptidases are primarily bound to the cell surface or found extracellularly, ISP2 may not have access to them after parasites are internalized.

In summary, we investigated disease progression and immune responses during the chronic phase of *L. major* infection by using a combination of genetically modified parasites, bioluminescence live imaging, and flow cytometry. This enabled us to unravel the role of ISP2 *in vivo* after low-dose intradermal infection in C57BL/6 mice. The data reported indicate that *L. major* ISP2 is important for the survival of *Leishmania* infection in the skin *via* modulation of monocyte recruitment and microbicidal potential and the development of moDCs.

## Supplementary Material

This article includes supplemental data. Please visit *http://www.fasebj.org* to obtain this information.

## Abbreviations

dLN: draining lymph node
FBS: fetal bovine serum
IRF: interferon regulatory factor
ISP: inhibitor of serine peptidase
moDC: monocyte-derived dendritic cell
MPO: myeloperoxidase
NE: neutrophil elastase
T_h_: T helper
WT: wild type
