# Supplementary material for: Inhibitor of serine peptidase 2 enhances Leishmania major survival in the skin through control of monocytes and monocyte-derived cells
Source: FASEB J. 2017 Nov 16;32(3):1315–27. doi: 10.1096/fj.201700797R (PMC5892728; doi:10.1096/fj.201700797R)
Supplement: Supplementary file 1 [file fj.201700797R.sf1.pdf]

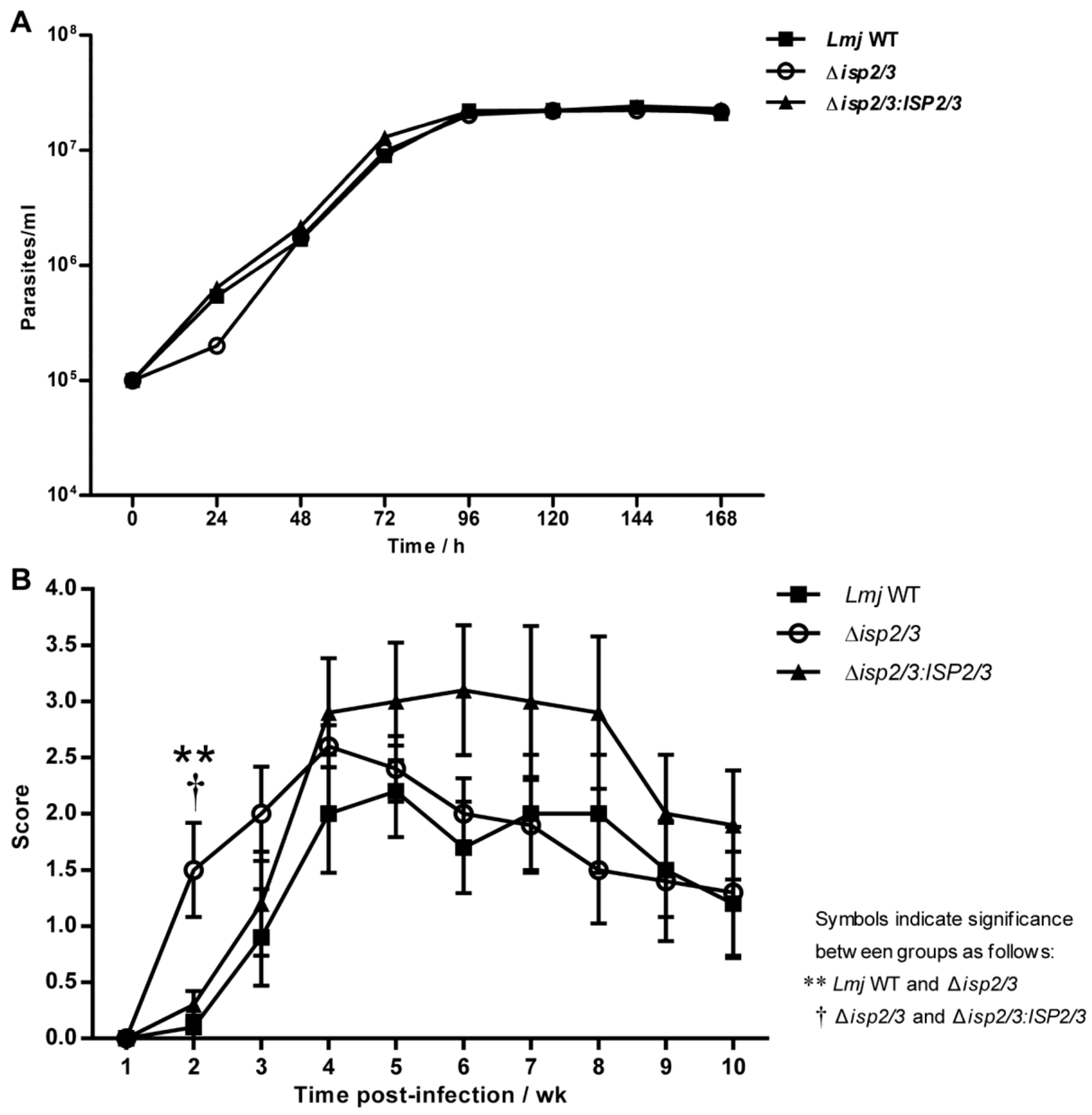

SUPPLEMENTAL FIGURE 1. *In vitro* growth and *in vivo* disease progression of *L. major* cell lines. (A) Promastigote cultures were started at 10^5 cells.ml^{-1} and cell densities were determined daily for 7d**. (B)** Ear lesion scoring of infected mice. C57BL/6 mice were inoculated in the ear with 10^4 *L. major* WT, Δ*isp2/3*, and Δ*isp2/3:ISP2/3* metacyclic promastigotes. Ear lesion scoring was measured weekly (n=5 for each group) and means ± SEM plotted. A representative graph of 2 independent experiments is shown. Symbols indicating statistical significance are as follows: ***Lmj* WT and Δ*isp2/3* ($P < 0.01$) and † Δ*isp2/3* and Δ*isp2/3:ISP2/3* ($P < 0.05$), as measured by one-way ANOVA with a Tukey post test.
