# Supplementary material for: Inhibitor of serine peptidase 2 enhances Leishmania major survival in the skin through control of monocytes and monocyte-derived cells
Source: FASEB J. 2017 Nov 16;32(3):1315–27. doi: 10.1096/fj.201700797R (PMC5892728; doi:10.1096/fj.201700797R)
Supplement: Supplementary file 2 [file fj.201700797R.sf2.pdf]

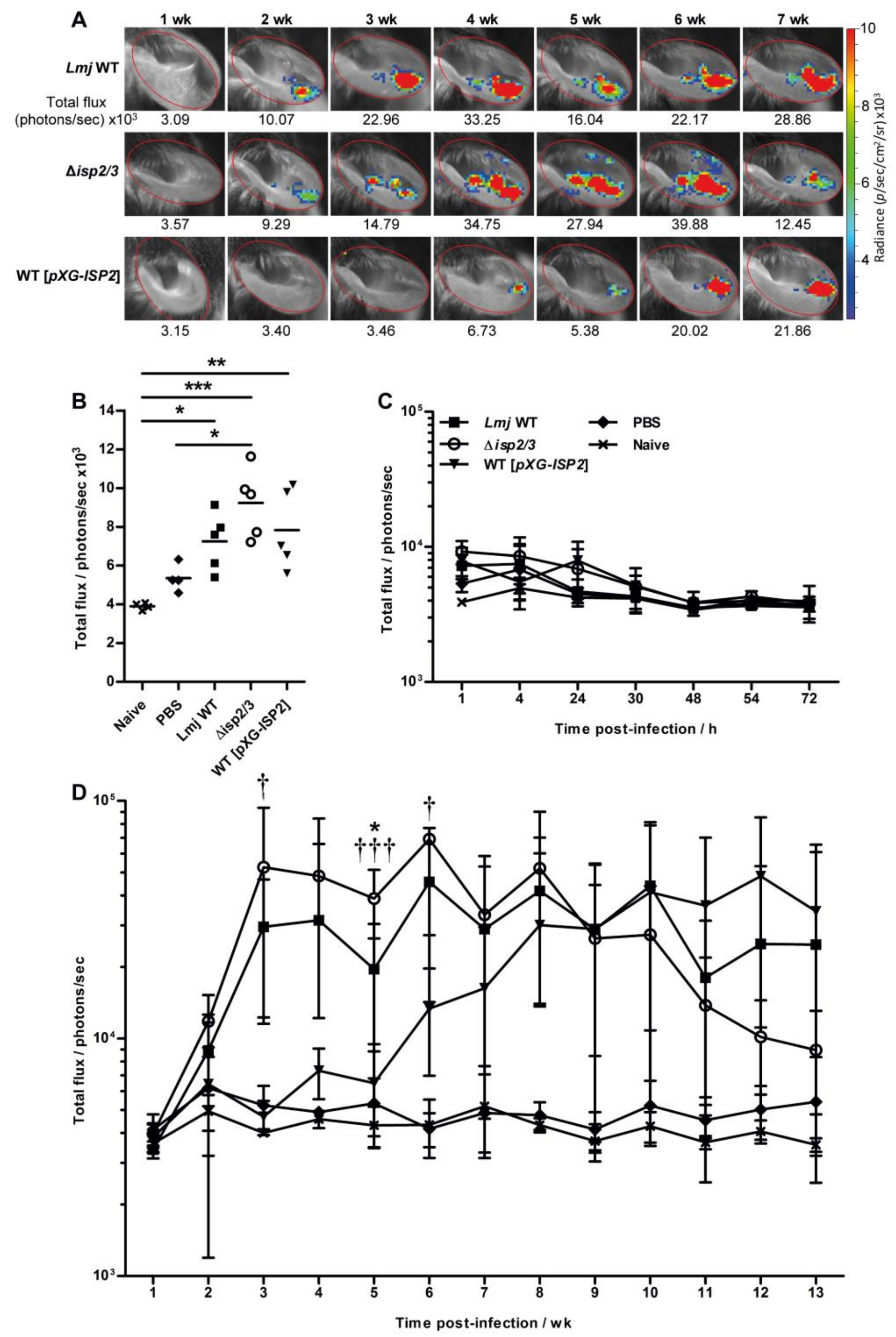

SUPPLEMENTAL FIGURE 2. *In vivo* bioluminescence imaging of MPO activity at the inoculation site during the early phase of infection with *L. major* WT and *ISP2* gene mutants. C57BL/6 mice were inoculated in the ears with 10^4 *L. major* WT, Δ*isp2/3*, and WT [*pXG-ISP2*] metacyclic promastigotes (n=5). The control groups were naive ears and ears injected with PBS (n=4 for each). Mice were imaged in the IVIS 10 to 15 min after intraperitoneal luminol injection. (*A*) Representative images of one mouse per group over 1-7 wk of infection for ears infected with parasite cell lines (as labelled on left). The color scale indicates bioluminescent radiance in photons/second/cm^2/steradian. The same color scale and region of interest (ROI, red oval) was applied to all images and the total flux for each ROI is given beneath the image. (*B*) The total flux over the ROI, given in photons per second (photons sec^{-1}), for each mouse 1 h after infection. Line indicates the mean. The mean total flux for the group at each time-point over (*C*) the first 72 h and (*D*) 1 to 13 wk of infection. Error bars represent SD. Symbols indicating statistical significance between the groups is as follows: *Lmj* WT and Δ*isp2/3:ISP2/3* at $P < 0.05$ (*) and Δ*isp2/3* and WT [*pXG-ISP2*] at $P < 0.05$ (†) and $P < 0.001$ (†††), as measured by one-way ANOVA with a Tukey post test.
