# Supplementary material for: Inhibitor of serine peptidase 2 enhances Leishmania major survival in the skin through control of monocytes and monocyte-derived cells
Source: FASEB J. 2017 Nov 16;32(3):1315–27. doi: 10.1096/fj.201700797R (PMC5892728; doi:10.1096/fj.201700797R)
Supplement: Supplementary file 3 [file fj.201700797R.sf3.pdf]

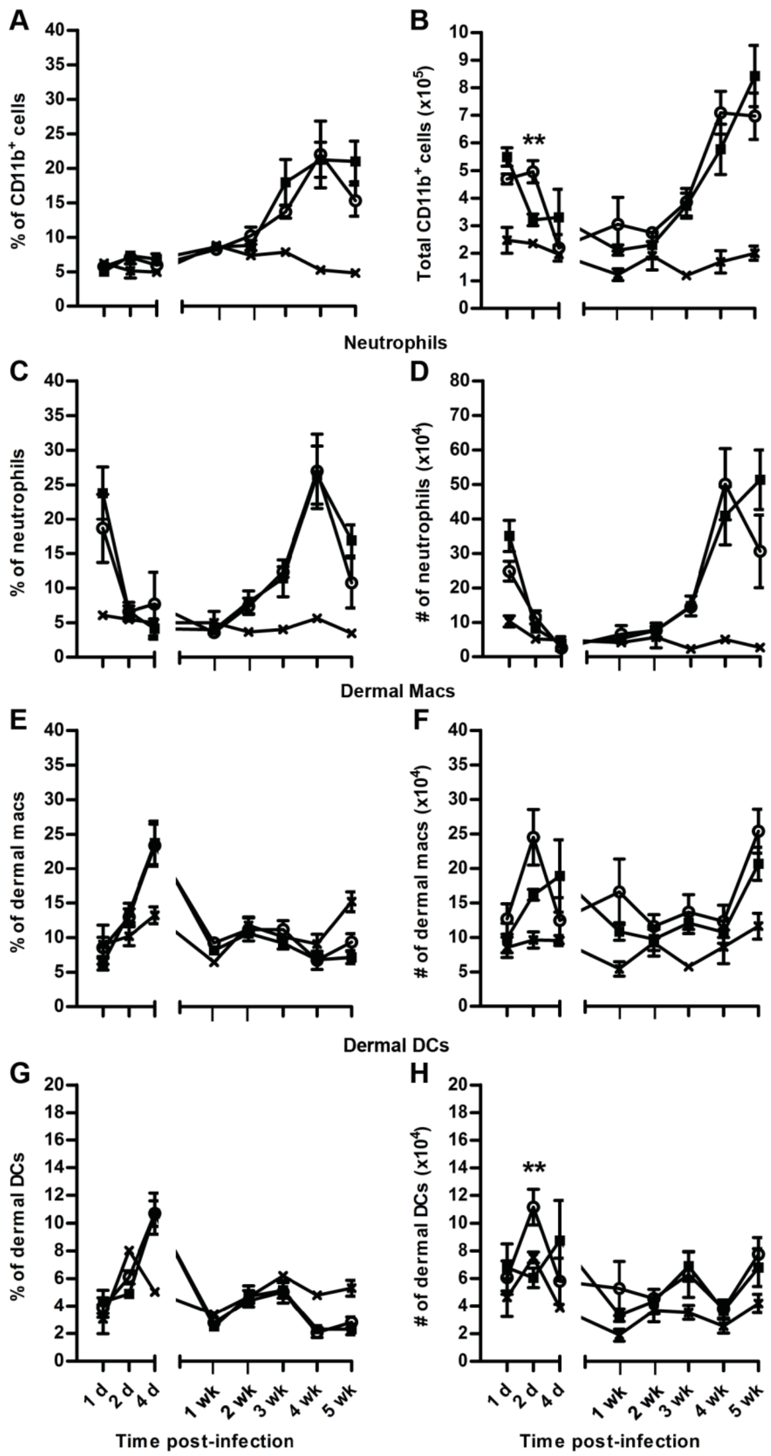

SUPPLEMENTAL FIGURE 3. Dynamics of the innate immune cell populations at the inoculation site during infection with *L. major* WT and Δ*isp2/3*. C57BL/6 mice were inoculated in the ears with 10^4 *L. major* WT (■) or Δ*isp2/3* (○) metacyclic promastigotes (n=5 for each time-point). Gating shown in Figure 4. (*A*) Percentage of $CD11b^+$ within the live cell population. (*B*) Changes in the total number of $CD11b^+$ cells. Changes in the percentages of each cell type within the $CD11b^+$ population (left panel) and changes in the total number of each cell type (right panels) per ear during infection. Neutrophils (*C* and *D*), dermal macrophages (*E* and *F*), and dermal dendritic cells (*G* and *H*). Naive ears from infected mice were used as a control at each time-point (×). Results are expressed as means per group at each time-point after infection, a representative of two independent experiments. Error bars represent SEM. Asterisks indicate statistical significance between WT and Δ*isp2/3* at $^{**}P < 0.01$, as measured by an unpaired *t*-test.
